# WOMEN SUPPORTING WOMEN: PEER SUPPORT GROUP FOR OLDER WOMEN WITH METASTATIC BREAST CANCER IN NIGERIA

**DOI:** 10.1093/geroni/igad104.1491

**Published:** 2023-12-21

**Authors:** Runcie Chidebe, Agha A Agha, Onyedikachi Nwakanma-Akanno, Chika Okem akwiwu, Candidus Nwakasi, Simeon Aruah, Darlingtina Esiaka, Phyllis Cummins

**Affiliations:** Miami University, Oxford, Ohio, United States; University of Nigeria Nsukka, Nsukka, Enugu, Nigeria; SMILE With Me Foundation, Lagos, Lagos, Nigeria; The Psych Lounge, Hoschton, Georgia, United States; University of Connecticut, Storrs, Connecticut, United States; National Hospital Abuja, Abuja, Federal Capital Territory, Nigeria; University of Kentucky, Lexington, Kentucky, United States; Miami University, Oxford, Ohio, United States

## Abstract

Metastatic breast cancer (mBC) is incurable and devastating to all cancer patients. In Nigeria, the proportion of patients with stage III or IV/metastatic breast cancer at diagnosis is 98%. Although many younger mBC patients live for years, older mBC patients have poorer prognosis, more physical symptoms, poorer access to palliative care, and experience higher social isolation. Also, most older Nigerian women diagnosed with mBC experience psychological challenges during and after their treatment, with higher possibility of abscondment from treatment. While peer support groups (PSG) have become increasingly beneficial for improving the quality of life (QoL) of these older women, there is paucity of literature exploring the benefit of these groups and how they provide support to cancer patients in Nigeria. Using a qualitative research approach and interpretative phenomenological analysis, we explored the experiences of older Nigerian women (n=8) living with mBC who belong to a PSG. Our finding shows that for older women living with mBC, seeing peers with similar lived experiences inspire them to keep living, being hopeful, and experience a reduction in death anxiety. PSG provides a culture of love, peer support, socialization and strengthened connections. In addition to pain and inability to walk (mBC linked disabilities) being recurring physiological challenges for many older women with mBC, forced retirement/inability to work, death anxiety, financial burden, and limited access to cancer care are some of the socio-psychological challenges they face. Therefore, the establishment of PSG in cancer treatment centers in Nigeria would improve QoL and reduce abscondment from treatment.

